# Perceived ankle instability and cutaneous reflex modulation during gait

**DOI:** 10.14814/phy2.15880

**Published:** 2023-11-23

**Authors:** Annalee M. H. Friedman, Leif P. Madsen

**Affiliations:** ^1^ Indiana University Bloomington Indiana USA

**Keywords:** chronic ankle instability, cutaneous reflexes, perceived instability

## Abstract

Cutaneous reflex modulation during rhythmic ambulation is an important motor control mechanism to help minimize stumbling following an unexpected perturbation. Previous literature found individuals with chronic ankle instability (CAI) experience altered reflex patterns compared to healthy controls. Considering CAI is characterized by intermittent feelings of ankle instability, researchers have speculated that these alterations are related to perceived instability. Our purpose was to determine whether variability and magnitude of cutaneous reflex amplitudes can predict perceived instability levels following sural nerve stimulation during gait. Forty subjects walked while receiving random stimulations and reported their perceived instability. Middle latency reflexes among lower leg muscles were calculated using data derived from surface electromyography. Hierarchical logistical regressions revealed a positive relationship between reflex variability of the peroneus longus and lateral gastrocnemius muscles and perceived instability during midstance. This suggests subjects with consistent reflexes following sural nerve stimulation develop a certain level of perceptual expectation resulting in generally lower feelings of ankle instability, while subjects with more variable motor outputs perceive greater instability at the supraspinal level. Cutaneous reflex variability during stance may be an important objective outcome measure to monitor neuromuscular recovery throughout a rehabilitation or as a potential predictor of future lateral ankle sprains.

## 1 INTRODUCTION

Cutaneous reflexes play an important role in maintaining coordinated movement during gait. Because human locomotion does not occur in a controlled environment, spinal reflexes are crucial for adaptation to environmental abnormalities such as uneven surfaces, physical obstacles, or sudden perturbation. During a cutaneous reflex arc, information from afferent receptors in the skin is processed in the spinal cord, modulating an appropriate motor response to continue a motor program, such as gait, or avoid injury. This may result in an increase (facilitation) or decrease (inhibition) of muscle activity compared to unperturbed movement which may or may not be associated with subsequent kinematic change. Utilizing polysynaptic pathways via interneurons, feedback from cutaneous stimulation can be modulated in accordance with the phase of the movement cycle, the task, or the intensity of the stimulation to maintain normal gait and avoid injury (Zehr & Stein, 1999).

A commonly observed example of phase‐dependent modulation during gait may be seen in the tibialis anterior (TA), where non‐noxious electrical stimulation to the sural nerve elicits facilitation during the early swing phase of gait, resulting in ankle dorsiflexion to lift the foot away from the ground to avoid tripping over an obstacle (Duysens et al., 1992; Madsen et al., 2020; Zehr et al., 1998). However, during late swing, the same stimulation results in an inhibition of the TA as a “placing response”, expediting the foot's contact with the ground to position the body in a more stable, double‐leg stance (Duysens et al., 1992; Madsen et al., 2020; Zehr et al., 1998). This response will also vary based on the demands of the task. For example, a study by Lamont, et al. found an enhancement of this TA “reflex reversal” during stair climbing compared to normal gait which was attributed to the increased stability threat posed by the task (Lamont & Zehr, 2006). During these rhythmic activities, cutaneous reflex modulation is thought to be coordinated by human central pattern generation (CPG) which integrates all sensory and positional information at the spinal and supraspinal levels to prompt an appropriate, interlimb motor response across muscle groups (Klarner & Zehr, 2018; MacKay‐Lyons, 2002; Zehr, 2005). Spinal‐level collaboration between cutaneous reflexes and CPGs is evidenced by bilateral reflexes following unilateral stimulation and coordinated upper and lower extremity reflexes during a variety of rhythmic activities (Haridas & Zehr, 2003; Lamont & Zehr, 2006; Sasada et al., 2010, 2021; Van Wezel et al., 1997; Zehr et al., 2004, 2009).

While cutaneous reflex modulation patterns are typically consistent among healthy individuals, recent research has identified discrepancies in reflexes among those with chronic ankle instability (CAI) (Futatsubashi et al., 2013, 2016, 2022; Heimark et al., 2023; Madsen et al., 2019). Manifesting in 40%–70% of individuals following a lateral ankle sprain, CAI is characterized by a variety of sensorimotor deficits and long‐term sequelae such as increased risk of re‐injury, early‐onset osteoarthritis, and subsequent lower overall health‐related quality of life. (Donovan et al., 2020; Hertel & Corbett, 2019; Thompson et al., 2018). When exploring sural nerve reflexes while in a seated position, a 2013 study by Futatsubashi, et al. identified greater inhibition of the peroneus longus (PL) and vastus lateralis among CAI subjects compared to controls (Futatsubashi et al., 2013). These reflexes occurred bilaterally, even when the previous injury occurred unilaterally, suggesting these variations stem from adaptations at the spinal or supraspinal level rather than damage to local afferents (Futatsubashi et al., 2013). Similar findings for the PL were replicated in a later study when investigating changes in cutaneous reflexes over time in subjects with a history of multiple ankle sprains (Futatsubashi et al., 2016). These findings suggest a lack of stability of the ankle and knee joints after sudden perturbation which may contribute to inadequate protection from injurious mechanisms. Considering the task‐dependent nature of cutaneous reflex modulation, recent studies in our lab have explored sural nerve reflexes to identify potential deficits in CAI reflex patterns during more functionally applicable tasks. During a drop‐landing task, Heimark et al. (2023) found those with CAI exhibited a lack of PL facilitation and resultant lack of eversion just prior to landing, indicating this group may be at a greater risk for sudden inversion upon contact with the ground (Heimark et al., 2023). Additionally, Madsen et al. (2019) found CAI subjects exhibited a lack of gastrocnemius inhibition when stimulated during the stance phase of gait. Since this inhibition is believed to be a protective response to unloading body weight onto the contralateral limb in healthy individuals, a lack of unloading may indicate those with CAI are less prepared to appropriately cope with unexpected perturbation during stance (Madsen et al., 2019; Zehr et al., 1998).

The latter two studies were conducted in our lab where we also identified anecdotal evidence linking altered cutaneous reflexes to specific CAI symptoms. First, the unique reflex patterns identified during the stance phase of gait were not universal among the CAI group which indicates certain subjects within this group were not experiencing the same type of impairments. The updated model of CAI put forth by Hertel and Corbett (2019) describes CAI as multifaceted, with each patient demonstrating a unique combination of neuromuscular deficits and subjective symptoms. This variability means the CAI groups in the studies previously discussed are likely not homogenous, therefore, before clinical application can be drawn from these findings, it is necessary to identify which impairments are associated with these altered reflexes. Second, during both gait and drop‐landing tasks, we noted several subjects reported that the sural nerve stimulations used for testing evoked a sensation similar to episodes of giving way at the ankle. While we know perceived instability is a hallmark symptom of CAI, its specific origins are not well understood and have been debated in the literature. Though mechanical deficits such as pathological laxity and impaired arthrokinematics were originally thought to be the basis for giving way at the ankle (Hertel, 2002; Hubbard & Hertel, 2006), more recent literature indicates this instability remains after initial tissue healing and may be indicative of sensorimotor maladaptation (Arnold & Docherty, 2006; Hertel, 2008; Sefton et al., 2009; Thompson et al., 2019). When defining CAI for research purposes, the International Ankle Consortium requires a history of ankle sprain and repetitive episodes or perceptions of instability at the ankle (Gribble et al., 2013). Not all individuals who experience ankle sprains develop CAI, therefore, perceived instability may be a more important outcome measure to explore in the context of the altered reflex patterns seen among CAI subjects.

Therefore, the present study sought to further explore the relationship between cutaneous reflexes and subjective feelings of ankle instability during gait. Specifically, we elicited non‐noxious electrical stimulations to the sural nerve at five equidistant time points of the stance phase of gait. Stance was chosen in the present study because, at this section of the gait cycle, people with a history of ankle sprains have been shown to experience altered cutaneous reflexes (Futatsubashi et al., 2016, 2022; Madsen et al., 2019) and subjective feelings of instability (Delahunt & Remus, 2019). Subjects were asked to rate their feelings of instability from 0 to 10 immediately following nerve stimulation. Additionally, average reflex amplitude and reflex variability across four lower leg muscles were calculated during offline analysis. Using hierarchical logistical regressions, we aimed to determine whether a person's ratings of ankle instability following sural nerve stimulation could be predicted by various independent variables including number of previous ankle sprains, presence of CAI, variability in cutaneous reflex amplitudes, and magnitude of cutaneous reflex amplitudes. We hypothesized that subjects with more variability in cutaneous reflex amplitudes across several stimulation trials would be among the subjects reporting higher levels of perceived ankle instability. If variability in cutaneous reflex amplitudes can predict perceived instability ratings, this outcome measure may be used to identify those at greater risk for intermittent feelings of instability during functional activities.

## 2 MATERIALS AND METHODS

### 2.1 Subjects

Forty subjects (12 male, 28 female) aged 20.2 ± 1.9 years were recruited for this study. All subjects were physically active, defined as ≥120 min of physical activity participation per week. Exclusion criteria included diagnosed neuromuscular conditions such as Parkinson's or multiple sclerosis, a history of fractures or surgeries in the lower extremity, and injury to the lower extremity within 6 weeks prior to data collection. After completing an informed consent document approved by the University's Institutional Review Board, all subjects completed a health history questionnaire (to rule out possible exclusion criteria) as well as the Identification of Functional Ankle Instability (IdFAI) and Foot and Ankle Ability Measure (FAAM) to identify appropriate test limbs. The language of the FAAM was modified slightly to reflect ankle instability rather than general function (see Appendix 1). To better understand the potential relationships between perceived instability, reflexes, and injury history, a broad sample of individuals was recruited, presenting with a variety of injury and instability characteristics. Based on IdFAI scores, this study included 15 subjects with CAI (IdFAI ≥ 11), 16 healthy controls (IdFAI = 0), and 9 subjects not fitting into either category (0 > IdFAI < 11) (Table 1). The “in‐between” individuals are those who either experienced a lateral ankle sprain (LAS) but do not experience instability (coper) or they experience instability but have not reported an acute ankle sprain. Each subject's test limb was identified based on their responses to the IdFAI and FAAM. In subjects with reported ankle instability, the most affected limb served as the test limb while controls were matched for limb dominance. Dominance was determined by asking with which limb the subject would kick a soccer ball. Three subjects reported greater instability on their non‐dominant limb, with 3 matched subjects reporting no instability, a total of 6 subjects were tested on their non‐dominant limb.

**TABLE 1:** Metrics used to evaluate the history of ankle injury and perceived instability across the entire cohort of 40 subjects which suggest an even representation of healthy control subjects, people with chronic ankle instability (CAI), and those who do not fit into either category (“in‐between”).

Subjects	Ankle sprains	IdFAI score	Modified FAAM score	Perceived instability post‐stimulation
Healthy control (*n* = 16)	0	0	100	2.64 ± 2.13
CAI (*n* = 15)	2.20 ± 1.21	16.9 ± 3.96	91.0 ± 6.45	3.16 ± 1.81
“In‐between” (*n* = 9)	1.78 ± 1.64	5.89 ± 3.68	97.7 ± 2.40	2.42 ± 1.64

Abbreviations: IdFAI, Identification of Functional Ankle Instability; FAAM, Foot and Ankle Ability Measure.

### 2.2 Electromyography

Electromyographic (EMG) measurements were collected via disposable Ag/AgCL surface electrodes (Biopac Systems, Inc., Goleta, CA) applied over the muscle bellies of the medial (MG) and lateral (LG) gastrocnemius, TA, and PL. After shaving (when applicable), lightly abrading, and cleaning the skin, two electrodes were applied approximately 2 cm apart over each muscle, ensuring no contact between electrodes measuring another muscle. Muscle bellies were identified by asking the subject to isometrically contract against resistance applied by the researcher. A grounding electrode was applied over the tibial tuberosity after similar skin preparation procedures. Electrical leads were connected to the electrodes and secured with medical tape over the electrode and through loops in the leads to limit movement artifacts. Leads were then connected to wireless transmitters which communicated with a Biopac MP160 recording system with EMG100C amplifiers (Biopac Systems, Inc.) to collect EMG data at a sampling rate of 2000 Hz.

### 2.3 Electrical stimulation

A reusable stimulating bar electrode (Ambu, Inc., Columbia, MD) with conductive gel was taped to the ankle just posterior to the lateral malleolus over the sural nerve. Leads were secured to the lateral leg and thigh with medical tape and looped through a hook and fastener belt around the subject's waist. A DS7A constant current stimulator (Digitimer North America, LLC, Ft. Lauderdale, FL) connected to a custom‐built latency device was used to administer stimulations. Perceptual threshold (PT) was first identified by increasing the stimulation amplitude from zero until the subject reported any sensation from the electrical impulse around the foot or ankle. Radiating threshold (RT) was then identified by increasing the amplitude until the area of sensation grew into the lateral foot and up into the lower leg. The subject was asked to report when the sensation no longer grew in area, only in intensity, which determined the RT for that subject. Final stimulation intensity for testing was first set as the RT multiplied by 2.5 which was then reduced in most subjects (*n* = 31) to ensure the impulse was non‐noxious and did not produce a withdrawal reflex. Withdrawal reflex was considered any visual evidence or verbal communication from the subject of involuntary movement of the lower extremity following stimulation. This resulted in an average final intensity of approximately 2.0× RT. Prior to beginning the experimental protocol, the researcher elicited several test stimulations to ensure the radiating area of the stimulation was maintained after lowering the stimulation to its final intensity for all subjects.

During a 5‐min warm‐up session walking on a Gait Trainer series 3 treadmill (Biodex Medical Systems Inc., Shirley, NY), 5‐pulse stimulation trains (pulse width of 1.0 ms) at 200 Hz were elicited to confirm the subject was still comfortable, and a withdrawal reflex was not present during gait. In order to administer stimulation across the 5 phases of stance accurately, each subject's gait cycle timing was identified as an average of 5 complete cycles (heel strike of test limb to next ipsilateral heel strike) in the latter half of the warm‐up period. The average cycle was then divided into 8 equal phases of the gait cycle starting from zero, the first 5 of which served as the latency parameters for test stimulations. Custom heel‐toe sensors inserted into both shoes were used to identify gait cycle timing. These sensors communicated with the custom‐built latency adjustment device. To administer a 5‐pulse train, the researcher held a trigger which would then activate the stimulator upon subsequent heel strike. Latency of this stimulation was manually adjusted according to the desired gait phase for each trial using the custom‐built device. Heel‐toe sensor and stimulation timing data were recorded via a Biopac MP160 recording system with a UIM100C amplifier (Biopac Systems, Inc.). Foot sensor and stimulation data displayed on Acqknowledge 5.0 software were visually inspected to confirm accurate stimulation timing prior to beginning the experimental protocol.

### 2.4 Protocol

Subjects walked on a treadmill at 4 km/h, a comfortable walking pace that has been used in previous research (Lamont & Zehr, 2006; Madsen et al., 2019; Zehr et al., 1997), for approximately 30 min with a 5‐min warm‐up period. As described above, this warm‐up was used to adjust stimulation intensity as needed in addition to confirming proper equipment set‐up and allowing the subject to normalize their gait pattern. During this period, subjects were instructed on how to report perceived instability during testing. When a stimulation was administered, subjects would report on a scale of 0 to 10 how much instability they perceived. A report of zero was described as “no instability” and “normal gait” and 10 was described as “feeling as if you may stumble or lose balance” and “need to hold onto the handrails”. Average perceived instability ratings following stimulation across the walking task may be found in Table 1. When starting the experimental protocol, subjects received ~50 random stimulations to the sural nerve (10 at each of the 5 phases of stance) which occurred approximately 5–10 steps apart. A trial was considered failed if the subject did not feel the stimulation, did not report their perceived instability immediately after stimulation, or if the stimulation timing was incorrect for the trial either due to equipment failure or researcher error in triggering the stimulation latency device. These trials were repeated, and walking continued until 10 trials were successfully completed for each phase. At the end of testing, equipment was removed, and subjects were dismissed from the laboratory.

### 2.5 Data processing

All data processing was completed using AcqKnowledge 5.0 software (Biopac Systems, Inc.). Raw EMG data were filtered at a low‐frequency cutoff of 50 Hz and a high‐frequency cutoff of 500 Hz. The root mean square was then derived for the smoothed signals for each muscle. Stimulated gait cycles labeled by phase (1–5) during testing were reviewed for step timing consistency and successful trials (at least 9 for each phase) were marked accordingly for analysis. Unstimulated gait cycles measured throughout testing were selected and labeled to ensure accurate values for each subject's background muscle activity. Trials falling 3 gait cycles before or after a stimulated trial were removed leaving 70–100 unstimulated trials which were then ensemble averaged for comparison to stimulated trials. To compare values directly, all waveforms were normalized as a percentage of the maximum EMG amplitude during unstimulated gait cycles (%MVC). For each muscle, stimulated and unstimulated waveforms were aligned at the point of heel strike using data from heel‐toe sensors. Unstimulated waveforms were then subtracted from stimulated waveforms to acquire the final reflex trace. Previous literature has shown modulation occurring during the middle latency reflex (MLR) approximately 80–120 ms post‐stimulation (Futatsubashi et al., 2013; Madsen et al., 2019; Zehr & Stein, 1999). For each muscle at each phase, mean amplitude values were extracted from the reflex waveform 80–120 ms after the last pulse in the stimulation train. Approximately 10 MLR values for each of the 4 muscles during stance phases (1–5) were extracted for each subject for statistical analysis.

### 2.6 Statistical analysis

Separate analyses were conducted at each of the 5 stance phases to explore reflex amplitudes of the 4 lower leg muscles and their relationship to perceived ankle instability. First, we assessed the reflex modulation patterns across our pooled subjects by using one‐sample *t*‐tests for each muscle at each phase. These 25 separate *t*‐tests were used to ensure the reflex amplitudes obtained for our subjects were statistically significant from zero and maintained patterns of facilitation and inhibition across stance consistent with those seen in previous literature. A Bonferroni adjustment was implemented to adjust the a priori alpha level given the large number of hypotheses tested. Thus, *t*‐tests were deemed statistically significant with *p*‐values less than or equal to 0.01. Next, at each of the five stance phases, we utilized a hierarchical (sequential) multiple regression to understand whether perceived instability following sural nerve stimulation can be predicted based on history of ankle sprain, presence of CAI, variability of reflex amplitudes between stimulation trials, and magnitude of reflex amplitudes averaged across stimulation trials. Each regression included three separate models. Table 2 lists the specific independent variables that were added to the hierarchical regression for each model at each phase. In general, the first model (A) explored whether a history of previous number of ankle sprains and the presence of CAI (IdFAI score ≥ 11) can predict whether someone experiences instability following sural nerve stimulation. The second model (B) was used to try and improve this prediction by adding four additional independent variables to the regression, which included the variability (standard deviation) of reflex amplitudes for each muscle across the 10 separate stimulation trials at that phase. The third and final model (C) added 4 more independent variables representing the mean reflex amplitude for each muscle, an average of the 10 reflex amplitudes for each of the 10 trials at that phase.

**TABLE 2:** Specific variables included in the three different models of each hierarchical multiple regression. One hierarchical regression was performed at each of the five phases, so the dependent and independent variables (aside from those in Model A) involved data obtained from stimulations elicited in the same phase.

	Independent variables added to model	Dependent variable
Model A	Number of previous ankle sprains Presence of CAI	Average perceived instability across 10 stance phase trials
Model B	3 SD of PL reflex across 10 trials 4 SD of TA reflex across 10 trials 5 SD of MG reflex across 10 trials 6 SD of LG reflex across 10 trials	
Model C	7 Average PL reflex amplitude 8 Average TA reflex amplitude 9 Average MG reflex amplitude 10 Average LG reflex amplitude	

Abbreviations: CAI, chronic ankle instability; LG, lateral gastrocnemius; MG, medial gastrocnemius; PL, peroneus longus; TA, tibialis anterior.

## 3 RESULTS

### 3.1 Reflex amplitudes and patterns

**FIGURE 1:**
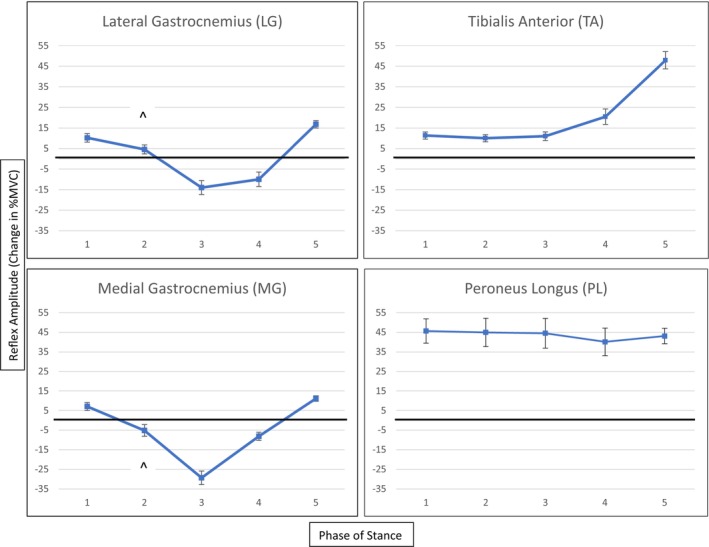
Average middle latency reflex amplitudes for 4 lower leg muscles across 5 phases of stance. Error bars represent the standard error of the mean. All reflexes were statistically significant from zero except for the medial gastrocnemius and lateral gastrocnemius at Phase 2 (indicated by the ^). Any values less than zero are indicative of muscle inhibition, while values greater than zero represent muscle facilitation.

Middle latency reflexes across the 40 subjects were first evaluated for the presence of extreme outliers by visually inspecting box and whisker plots. Only 4 outliers were identified across 2 muscles at 3 different phases. Specifically, one subject had abnormally high facilitation of the MG at phase 1, another subject presented with heightened inhibition of the MG at phase 4, and two separate subjects had abnormally high facilitation of the TA at phase 4. The outliers were left in the final analysis because they were obtained from true EMG reflex traces (not measurement errors), they were measured across four different subjects who were not outliers for any other muscle or phase, and running the *t*‐tests with and without the outliers in the analysis did not change the final interpretation of the results. The separate *t*‐tests found that the average reflex amplitudes were statistically significant from zero for all muscles at each phase (*p* < 0.01), except for the MG (*t*_(39)_ = −1.737, *p* = 0.09) and LG (*t*_(39)_ = 2.126, *p* = 0.04) at phase 2. Figure 1 shows the reflex amplitude patterns across the stance phases for each muscle.

### 3.2 Predicting perceived instability post‐stimulation

Perceived ankle instability following sural nerve stimulation remained consistent across all five phases of stance with no evidence of outliers. No subjects reported perceived instability higher than 8/10 for any phase, and the average instability for all subjects was between 2/10 and 3/10 for all phases. The hierarchical multiple regressions were then performed to determine whether the three different models were statistically significant in predicting these perceived levels of instability.

#### 3.2.1 Model A

The Hierarchical multiple regressions at each phase began by investigating whether having a history of ankle sprains or having CAI can help predict perceived ankle instability post‐sural nerve stimulation. The regression model including only these two independent variables was not able to statistically predict perceived instability at any of the 5 phases (*p* > 0.05).

#### 3.2.2 Models B and C

The addition of reflex variability (Model B) and reflex amplitude (Model C) independent variables did not significantly improve the prediction of perceived instability for phases 1, 2, 4, and 5 (*p* > 0.05). However, for phase 3 (representing the midstance phase), Model B did reach statistical significance, *R* ^2^ = 0.421, *F*(6, 33) = 4.005, *p* = 0.004, adjusted *R* ^2^ = 0.316. More specifically, the addition of standard deviation variables from the 4 lower leg muscles to the prediction of instability ratings led to a statistically significant increase in *R* ^2^ of 0.367, *F*(4, 33) = 5.226, *p* = 0.002. Further addition of average reflex amplitude variables (Model C) was also statistically significant, *R* ^2^ = 0.512, *F*(10, 29) = 3.045, *p* = 0.009, adjusted *R* ^2^ = 0.344. However, the change in *R* ^2^ from Model B to Model C was only 0.091 and was not statistically significant (*F*(4, 29) = 1.351, *p* = 0.275) suggesting variability in reflexes among these 4 lower leg muscles is more predictive of perceived instability ratings compared to average reflex amplitudes. Thus, we consider Model B as more representative of the final model for the phase 3 regression since average reflex amplitude variables can be removed without reducing the acceptable level of prediction.

#### 3.2.3 Phase 3 model coefficients

**FIGURE 2:**
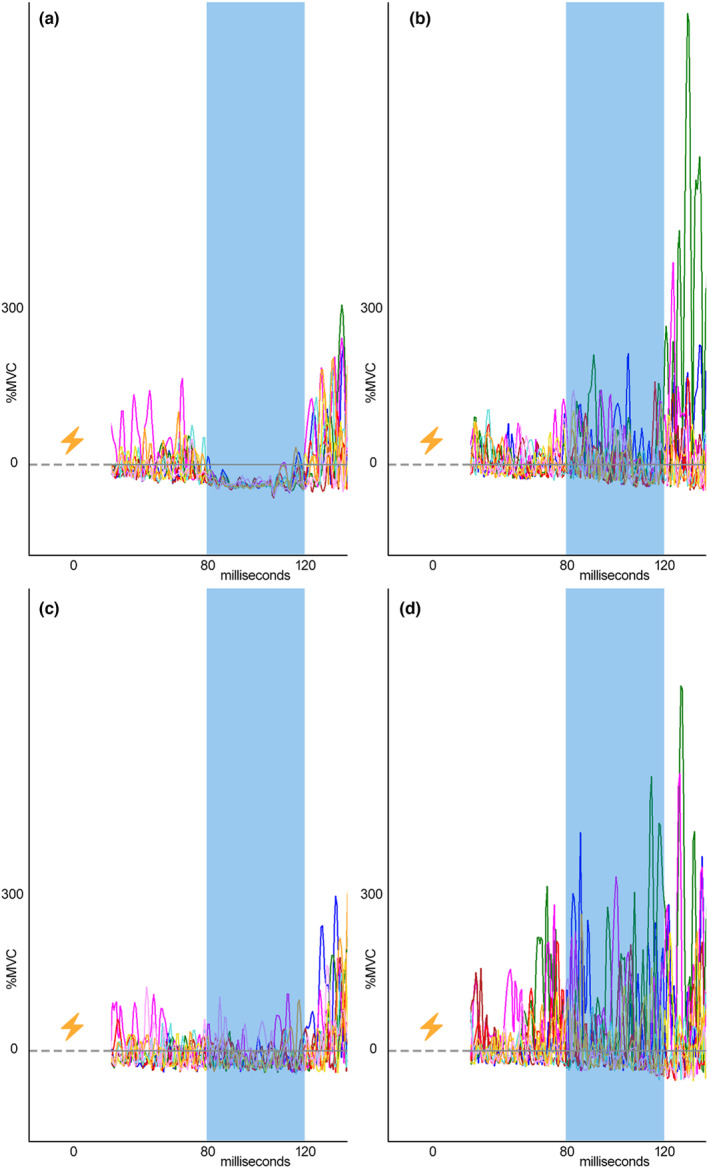
Example lateral gastrocnemius (LG) and peroneus longus (PL) net reflex traces for all trials with stimulation artifact removed (dotted line), middle latency reflex indicated by blue shaded area. (a) LG reflexes in a participant reporting “low” instability (*M* = 0.78/10). (b) LG reflexes in a participant reporting “high” instability (*M* = 6.67/10). (c) PL reflexes in the participant reporting “low” instability. (d) PL reflexes in the participant reporting “high” instability.

Since the Model B regression analysis reached statistical significance for phase 3, we further explored its model coefficients to identify specific variables within the prediction model that were most helpful in predicting the dependent variable. Table 3 provides the coefficients of Model A and Model B for this phase 3 multiple regression. The independent variables with significant slope coefficients in Model B include number of ankle sprains (*p* = 0.037), standard deviation of LG reflexes (*p* = 0.029), and standard deviation of PL reflexes (*p* = 0.005). Since the slope coefficient for the LG and PL variables is positive, this means that a one unit change of perceived instability (more feelings of instability) is associated with an increase in LG and PL reflex variability. Figure 2 illustrates this relationship in two example subjects. Additionally, the slope coefficient for ankle sprains is positive, suggesting that among the subjects with more reflex variability, those who have a history of ankle sprains have higher levels of perceived instability.

**TABLE 3:** Hierarchical multiple regression (models A and B) predicting perceptions of ankle instability following sural nerve stimulation at midstance (phase 3). Model C was excluded from this table as the change in *R* ^2^ was not statistically significant from Model B.

Independent variable	Perceived instability rating			
	Model A		Model B	
	*B*	*β*	*B*	*β*
Constant	2.24**		−0.307	
Number of ankle sprains	0.126	0.094	0.490*	0.363
Presence of CAI	0.687	0.171	−0.217	−0.054
SD of PL			0.045*	0.426
SD of TA			0.035	0.214
SD of LG			0.075*	0.340
SD of MG			−0.057	−0.204
*R* ^2^	0.055		0.421	
*F*	1.073		4.005*	
Δ*R* ^2^	0.055		0.367	
Δ*F*	1.073		5.226**	

*Note*: The coefficients within our final regression model for phase 3 (Model B) can be used to create a final prediction equation as follows: Perceived instability rating = −0.307 + (0.490 × Number of ankle sprains) − (0.217 × Presence of CAI) + (0.045 × SD of PL) + (0.035 × SD of TA) + (0.075 × SD of LG) − (0.057 × SD of MG). The “Presence of CAI” variable is dichotomous with a value of 1 denoting the presence of CAI and 0 representing no CAI.

Abbreviations: CAI, chronic ankle instability; LG, lateral gastrocnemius; MG, medial gastrocnemius; PL, peroneus longus; TA, tibialis anterior.

* *p* < 0.05;

** *p* < 0.01.

## 4 DISCUSSION

### 4.1 Reflex patterns during stance

Though our sample included both those with and without a history of ankle sprains, the net reflex patterns revealed by *t*‐tests aligned with those previously reported in healthy individuals. As mentioned earlier, CPGs are thought to play an integral role in coordinating cutaneous reflex modulation at the spinal and supraspinal levels throughout the gait cycle with the goal of maintaining a smooth stride and avoiding stumbling or injury following a perturbation. Therefore, the responses seen during the 5 phases of stance will vary depending on the specific position of the body during sural nerve stimulation. Considering the stimulation intensity used for this study was non‐noxious, the elicited perturbations would be interpreted as less threatening, and the goal of the reflex in each phase would be to continue the gait cycle as smoothly as possible. During heel‐strike (phase 1), subjects exhibited facilitation of the TA and PL which has been reported in previous studies as a response to dorsiflex and evert the ankle, respectively (Duysens et al., 1992; Madsen et al., 2019; Zehr et al., 1998). In the presence of an obstacle along the lateral portion of the foot, dorsiflexion would delay planting of the foot during heel strike and eversion would allow for pushing away from or against the obstacle, thus stabilizing the foot and avoiding potential inversion injury (Madsen et al., 2019; Zehr & Stein, 1999). Gastrocnemius facilitation was also exhibited which, in combination with TA and PL facilitation, would result in a bracing response as the foot contacts the ground, enhancing joint stability upon weightbearing.

In midstance (phases 2–4), our sample exhibited inhibition of the LG and MG, which is considered a preparatory response for potential transition to the contralateral limb in the case of a dangerous perturbation or loss of balance (Duysens et al., 1992; Madsen et al., 2020; Zehr et al., 1998). Facilitation of the TA and PL during these phases would contribute to the unloading response seen in the plantarflexors to, again, allow for an efficient transition to the contralateral limb should the perturbation become dangerous or affect postural stability. At toe‐off (phase 5) this facilitation increased in the TA which would generate further dorsiflexion to quickly to lift the foot over the obstacle to avoid stumbling and continue into swing. Madsen et al. (2019) also reported a large TA facilitation during phase 5 among both CAI and control groups which resulted in significant net dorsiflexion. The PL facilitation observed at toe‐off in our present sample would act to evert and push away from an obstacle as the limb begins the transition to swing and has been reported among both healthy and CAI subjects of previous studies (Madsen et al., 2019, 2020). Significant facilitation of the LG and MG was observed during this phase as well, allowing for a more rapid transition to the swing phase through plantarflexion which pushes the ipsilateral limb off the ground and subsequently shifts weight to the contralateral limb in the presence of an obstacle (Madsen et al., 2019; Zehr et al., 1998). The gastrocnemius muscles' role as knee flexors may also assist in this transition to further lift the ipsilateral limb off the ground in tandem with dorsiflexion resulting from the aforementioned TA facilitation.

### 4.2 Reflex variability and perceived instability

While previous literature exploring cutaneous reflexes has focused on average reflex amplitudes, the results of our analyses indicate reflex variability may be a more accurate indicator of sensorimotor maladaptation following ankle injury. During phase 3 (midstance) previous studies reported inhibition of the LG as a protective unloading response in healthy individuals, but no significant reflex was identified in those with CAI (Madsen et al., 2019, 2020). Considering the multifaceted nature of CAI, we may suggest that not all subjects with this condition will exhibit altered reflex patterns. The small sample (*n* = 8) used in Madsen et al. (2019) may have exhibited enough variability in the LG reflex across 10 trials in this phase to affect the statistical significance, resulting in the lack of LG inhibition reported for this group. The sample size used in the present study (*n* = 40) is ample to draw clearer conclusions about the reflex characteristics of healthy and CAI subjects, alike. While our sample collectively exhibited LG inhibition at this phase when averaged, the high variability of these responses indicates not all stimulation trials resulted in consistent reflex amplitudes for each subject. One potential reason why a subject may experience a high variability in cutaneous reflexes from trial to trial is simply due to fluctuations in background muscle activity. Due to automatic gain compensation, any trial in which a subject has higher voluntary muscle activity immediately prior to the stimulation could certainly result in a more pronounced reflex (Matthews, 1986). However, after exploring the background EMG activity 20 ms prior to stimulation across our subject pool, we found that the variation of muscle activity between stimulation trials was consistent between the low‐instability and high‐instability subjects with a standard deviation of approximately 8% MVC. Interestingly, despite having similar fluctuations in background EMG activity across the 10 trials, subjects were still experiencing significantly different variability of reflex amplitudes. This observation suggests that the large variability in reflexes among our high‐instability subjects is more related to unique sensorimotor control mechanisms rather than inconsistent muscle activity while walking.

Considering the protective purpose of cutaneous reflex modulation patterns, departure from the appropriate response, even occasionally, following a perturbation to the lateral foot or ankle would leave individuals more vulnerable to stumbling or injury. The relationship between perceived instability and reflex variability identified in the LG and PL during midstance indicates greater inconsistencies in motor output following perturbation may result in greater perceptions of ankle instability. Regardless of the average reflex amplitudes measured across several stimulation trials in this study, the dispersion in these amplitudes paints a more complete picture of the actual neuromuscular responses seen in individuals reporting various levels of instability during pertured gait. Individuals with little to no perceived instability exhibit consistent reflex amplitudes, indicating the response to stimulation is adequate and, thus, does not provoke cognitive distress. Meanwhile, those reporting higher levels of instability following stimulation exhibit more variability in reflex amplitudes, some of which may be inadequate for protection against stumbling or injury should perturbation continue. Our results also indicate that in combination with higher reflex variability, a history of ankle sprains also contributes to higher levels of perceived instability. A 2016 study exploring cutaneous reflexes in individuals recovering from ankle sprains found PL MLRs were closely associated with functional recovery of the ankle (Futatsubashi et al., 2016). Specifically, subjects with only one previous sprain recovered within 3 months while those with 2 or more sprains exhibited persistent reflex deficits during an isometric eversion task (Futatsubashi et al., 2016). This supports the present findings that while recurrent sprains are not a predictor of perceived instability (Model A), they may contribute to reflex deficits and associated perceived instability following sural nerve stimulation.

In addition to the results of the present study, previous literature provides evidence of a relationship between perceived instability and spinal‐level alterations. Harkey et al. (2016) identified a quadratic association between neural excitability and self‐reported function via FAAM scores among those with CAI. That is, subjects exhibiting low or high soleus spinal‐reflexive and corticomotor excitability presented with lower overall perceived foot and ankle function. The authors elaborate on the functional implications of these reflex variations, specifically, that low levels of neural excitability of the soleus would result in decreased postural control or poor stabilization of the ankle during gait while too much neural excitability may create excessive plantarflexion, perpetuating the sensation of “giving way” (Harkey et al., 2016). Though not directly related to perceived instability, the FAAM quantifies the level of difficulty an individual experiences when participating in a variety of activities and, thus, may include instability as well as other outcome measures such as balance or strength deficits as contributors. Additionally, Thompson et al. (2019) found that perceived instability reported via the Ankle Instability Instrument and Cumberland Ankle Instability Tool could predict 20% of the variance in soleus spinal‐reflexive excitability among those with CAI. These questionnaires include instability‐specific items and, therefore, may be a more direct comparison to the findings of the present study. Interestingly, this relationship was identified when subjects were in single‐leg stance which, while not a CPG‐driven dynamic task, is comparable to the midstance phase of gait (Thompson et al., 2019). The authors also identified those with CAI exhibited a disinhibition of the soleus, indicating an inability to properly modulate spinal reflexes during tasks that threaten postural stability.

### 4.3 Clinical application

The results of this study suggest little correlation between CAI status as determined by IdFAI scores and perceived instability following sural nerve stimulation. However, when evaluating other specific objective outcome measures, our results suggest reflex variability may predict perceived instability following stimulation. Identifying a specific biomarker in all individuals regardless of CAI status allows for the treatment of that specific deficit directly as well as its subsequent symptoms. Since sural nerve stimulation is intended to mimic an obstacle contacting the lateral surface of the foot and ankle, identifying subjects' levels of instability following each stimulation offers insight as to how an individual may feel when coming across an obstacle in an uncontrolled environment. Perceived instability is typically intermittent rather than occurring during every bout of physical activity and is often reported during specific tasks that require more frontal plane movement or in environments with uneven surfaces (Attenborough et al., 2014; Donovan et al., 2020). Though this symptom is not consistent, the unpredictable nature of perceived instability likely contributes to the fear of re‐injury and subsequent reduced physical activity over time seen in individuals with CAI (Houston et al., 2014; Hubbard‐Turner & Turner, 2015; Suttmiller & McCann, 2021). From both a patient and public health perspective, it is important to curtail these sequelae as they contribute to poorer overall health‐related quality of life and incur financial burdens (Feger et al., 2017; Houston et al., 2014). Terada et al. (2017) further explored the concept of perceived instability, finding sensorimotor components of CAI including postural control and neural excitability were good predictors of CAI for subjects experiencing both feelings of instability and recurrent sprains, but could not predict perceived instability alone (Terada et al., 2017). The authors indicated the condition may simply be too heterogeneous to categorize and individuals with CAI should be treated based on their specific symptoms (Terada et al., 2017). The present findings indicate reflex variability may serve as an objective outcome measure to identify and monitor sensorimotor recovery. Identifying the specific limitations that comprise an individual's unique symptoms allows the clinician to effectively treat deficits through either traditional neuromuscular therapies or even direct stimulation interventions, many of which have been explored in the context of enhancing neural output or functional outcome measures such as joint position sense (Migel & Wikstrom, 2021; Pearcey & Zehr, 2020; Ross et al., 2015; Sharma et al., 2020). Like other common measures, such as balance or proprioception, altered reflexes may be targeted to treat each patient based on their specific deficits. Additionally, these findings may indicate sural nerve stimulations during gait could be used to elicit feelings of instability in a controlled environment, a symptom that is difficult to replicate due to its intermittent nature. By observing reflexive characteristics and perceptions of instability following cutaneous stimulation, we may gain better insight into how these sensorimotor deficits manifest and change over the course of a rehabilitation program.

### 4.4 Limitations and future directions

A limitation of the present study was the inherently subjective nature of perceived instability. The researchers sought to control this variable by describing the rating scale to all subjects using the same script. A rating of 0/10 was described as no instability experienced and a 10/10 was described as the participant feeling they felt they may stumble or fall off the treadmill. Because the sural nerve stimulations were non‐noxious, it was rare that any subject reported on the higher end of the instability scale, and all subjects individually reported consistent levels of instability across stimulated trials with a standard deviation ranging from 1.85–2.05 throughout stance. It should be noted that we do not know whether altered reflexes are the cause or effect of perceptions of instability. Our current findings cannot explain why some subjects with no history of ankle sprains or instability still reported feelings of instability following sural nerve stimulation. Previous literature has suggested the pathways that mediate pain, anxiety, or perceived instability affect motor output, revising a muscular response based on specific sensory input associated with the task and learned modulatory programs at the spinal or supraspinal level (Sibley et al., 2007; Thompson et al., 2019). Exploration of perceived instability and reflex variability should be continued among healthy individuals as well as those with recurrent ankle sprains to better understand the nature of this relationship. In addition to monitoring perceptions of instability and reflex variability in the CAI population throughout rehabilitation, individuals with no history of ankle injury could also be monitored to determine if their altered reflexes make them more susceptible to future LAS.

In the present study, we did not measure thigh musculature or musculature on the contralateral limb. Previous literature has shown that BF and VL also contribute to cutaneous reflex modulation during the stance phase of gait in healthy individuals to increase joint stiffness and reduce stumbling risk (Lamont & Zehr, 2006; Tax et al., 1995; Zehr et al., 1998). Additionally, considering the evidence for spinal level control of cutaneous reflex modulation via CPG, we can be confident that stimulation to one limb will likely result in modulation bilaterally (Dietz et al., 2001; Klarner & Zehr, 2018; Tax et al., 1995). Exploring cutaneous reflexes in bilateral musculature of the knee, hip, or upper extremity in the context of perceived instability would reveal the extent of this relationship in both healthy and CAI populations.

## 5 CONCLUSIONS

The results of this study indicate variability in PL and LG reflexes during midstance of gait predict feelings of instability following sural nerve stimulation. Inconsistent motor output following sudden perturbation could leave individuals more susceptible to stumbling or injury during functional activities, which may explain associated perceptions of instability. The diversity of the subjects experiencing perceived instability during testing indicates this subjective outcome measure may be more associated with reflex variability than CAI itself, though a history of ankle sprains does contribute to this relationship. Measuring cutaneous reflex variability may be important in identifying sensorimotor deficits contributing to CAI as well as treating frequent bouts of perceived instability during functional activities. Future research should identify how abnormal reflex characteristics may be used to monitor neural recovery from previous LAS and how interventions may be used to regulate cutaneous reflexes during functional activities.

## AUTHOR CONTRIBUTIONS

All experiments were performed in the Department of Kinesiology's Motor Control laboratory within Indiana University's School of Public Health. All authors contributed to the conception of this work, the collection and interpretation of data for the work as well as writing and critical revision of the work. All authors have approved the final version of this manuscript and agree to be accountable for all aspects of the work in ensuring that questions related to the accuracy or integrity of any part of the work are appropriately investigated and resolved. All persons designated as authors qualify for authorship, and all those who qualify for authorship are listed.

## CONFLICT OF INTEREST STATEMENT

All authors certify that they have no affiliations with or involvement in any organization or entity with any financial interest or non‐financial interest in the subject matter or materials discussed in this manuscript. No sources external funding were received for this research.

## ETHICS STATEMENT

All procedures performed in studies involving human subjects were in accordance with the ethical standards of the institutional and/or national research committee and with the 1964 Helsinki Declaration and its later amendments or comparable ethical standards. The presented study received ethical approval from Indiana University's Institutional Review Board prior to subject recruitment and written informed consent was obtained from all individuals participating in the study.

## Data Availability

The data that support the findings of this study are available on request from the corresponding author.
